# Palliative RVOT Stenting Using a Drug‐Eluting Coronary Stent in a Preterm Neonate With Severe PS, VSD, and Bilateral Branch PA Stenosis: A Case Report

**DOI:** 10.1002/ccr3.71637

**Published:** 2025-12-07

**Authors:** Reza Derakhshan, Mahsa Shadman, Najmeh Soltani Nejad

**Affiliations:** ^1^ School of Medicine Kerman University of Medical Sciences Kerman Iran; ^2^ Clinical Research Development Unit, Afzalipour Hospital Kerman University of Medical Sciences Kerman Iran; ^3^ Department of Pediatrics, Afzalipour Faculty of Medicine Kerman University of Medical Sciences Kerman Iran

**Keywords:** coronary stent, genetic disorder, palliative therapy, preterm neonate, RVOT stenting, tetralogy of Fallot

## Abstract

Early RVOT stenting using a drug‐eluting coronary stent can be a lifesaving palliative option in preterm neonates with critical RVOT obstruction. This minimally invasive approach improves pulmonary blood flow, supports pulmonary artery growth, and safely bridges high‐risk infants to definitive repair.

## 1 Introduction

Congenital heart disease (CHD) affects approximately 8–10 per 1000 live births [1]. Tetralogy of Fallot (TOF), the most common cyanotic CHD (13,500 births), is defined by VSD, overriding aorta, RVOTO, and right ventricular hypertrophy [2, 3]. Although most TOF remain asymptomatic during the early postnatal period, a subset of patients presents with significant clinical symptoms requiring early intervention.

The severity of RVOTO plays a decisive role in the clinical presentation and therapeutic decision‐making in affected neonates [4]. Primary surgical repair is the treatment of choice for most patients, as it improves oxygen saturation and promotes normal growth of the RVOT and pulmonary arteries. However, in certain neonates with comorbid conditions such as prematurity, low birth weight, complex cardiac anomalies, severe RVOT obstruction, or pulmonary artery hypoplasia or atresia—all of which are predictors of high‐risk surgical outcomes—early palliative intervention is recommended [5, 6, 7]. Thus, percutaneous RVOT stenting has emerged as a minimally invasive alternative and a bridge to definitive treatment.

## 2 Case Presentation

### 2.1 Patient Information

A female neonate from a 33‐year‐old mother (G5P4L2A1D2) was born at 36 weeks of gestation (LMP) via vaginal delivery, weighing 1700 g. A family history revealed two prior neonatal TOF‐related deaths, one preceded by catheterization. Apgar scores were six at 1 min and eight at 5 min.

## 3 Clinical Findings

At birth, the neonate had central cyanosis (preductal SpO_2_ 60%) and respiratory distress, requiring immediate intubation. On examination: tachypnea, subcostal retractions, cyanotic lips, and a systolic ejection murmur, and a holosystolic murmur along the left sternal border. No other anomalies were detected.

## 4 Diagnostics

Echocardiography at the first hours of birth identified:

Critical valvular and subvalvular pulmonary stenosis with RVOT tortuosity and nearly absent.

Antegrade flow (functionally near‐atresia).

Small perimembranous VSD and ASD.

Patent ductus arteriosus.

Severe bilateral branch pulmonary artery stenosis with hypoplastic RPA and LPA measuring approximately 1.5–2.0 mm, and MPA ~2.5–3.0 mm.

Severe tricuspid regurgitation.

## 5 Therapeutic Intervention

Initial stabilization included:

PGE_1_ infusion (0.02 μg/kg/min at first and after four hours increased to maximum dose).

Milrinone (0.5 μg/kg/min) and dopamine (5 μg/kg/min).

Propranolol, Empiric antibiotics, TPN.

**FIGURE 1:**
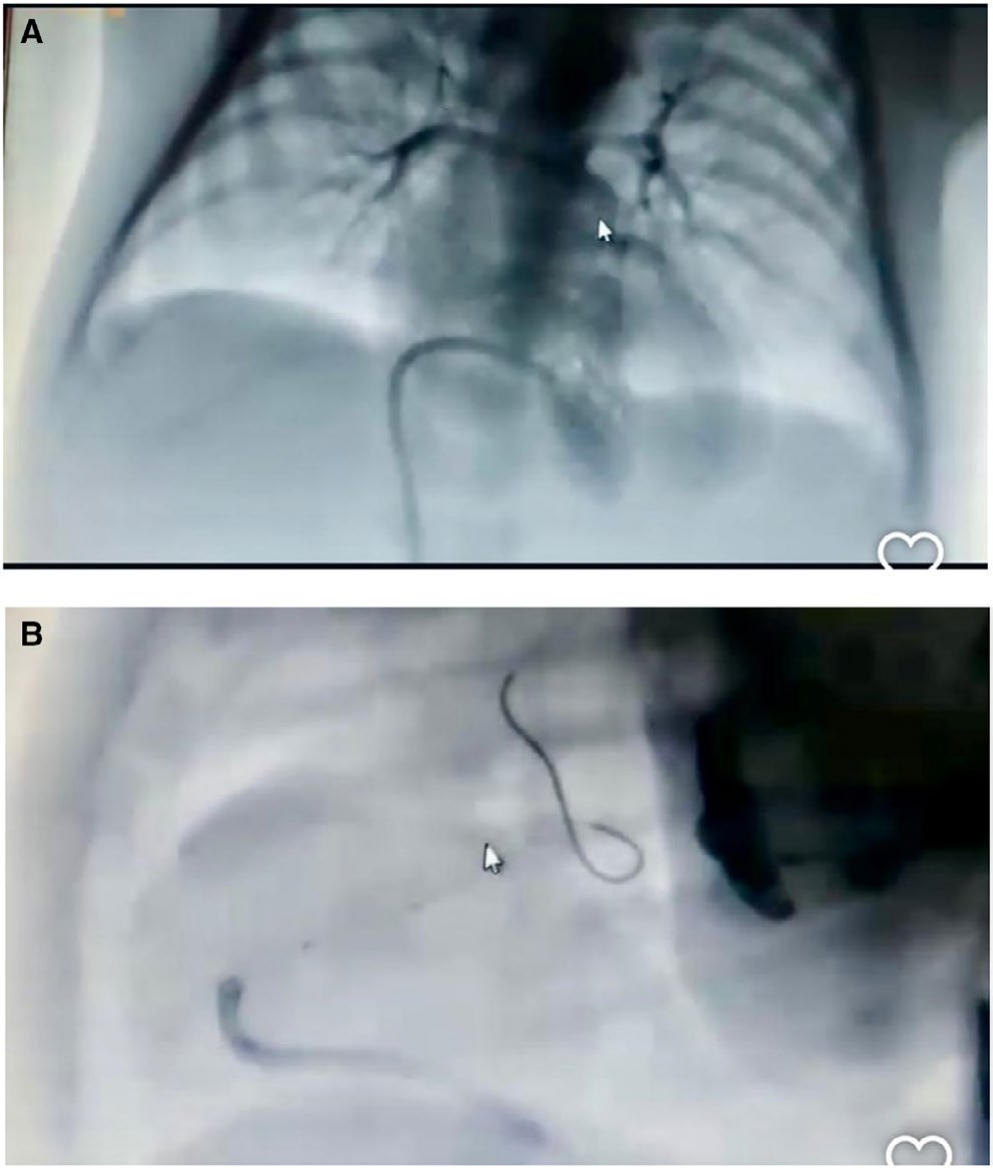
(A and B) Pre‐stent injection in AP and lateral views, revealing narrow branches of LPA and RPA, RVoT tortuosity, and multiple stenoses in it.

**FIGURE 2:**
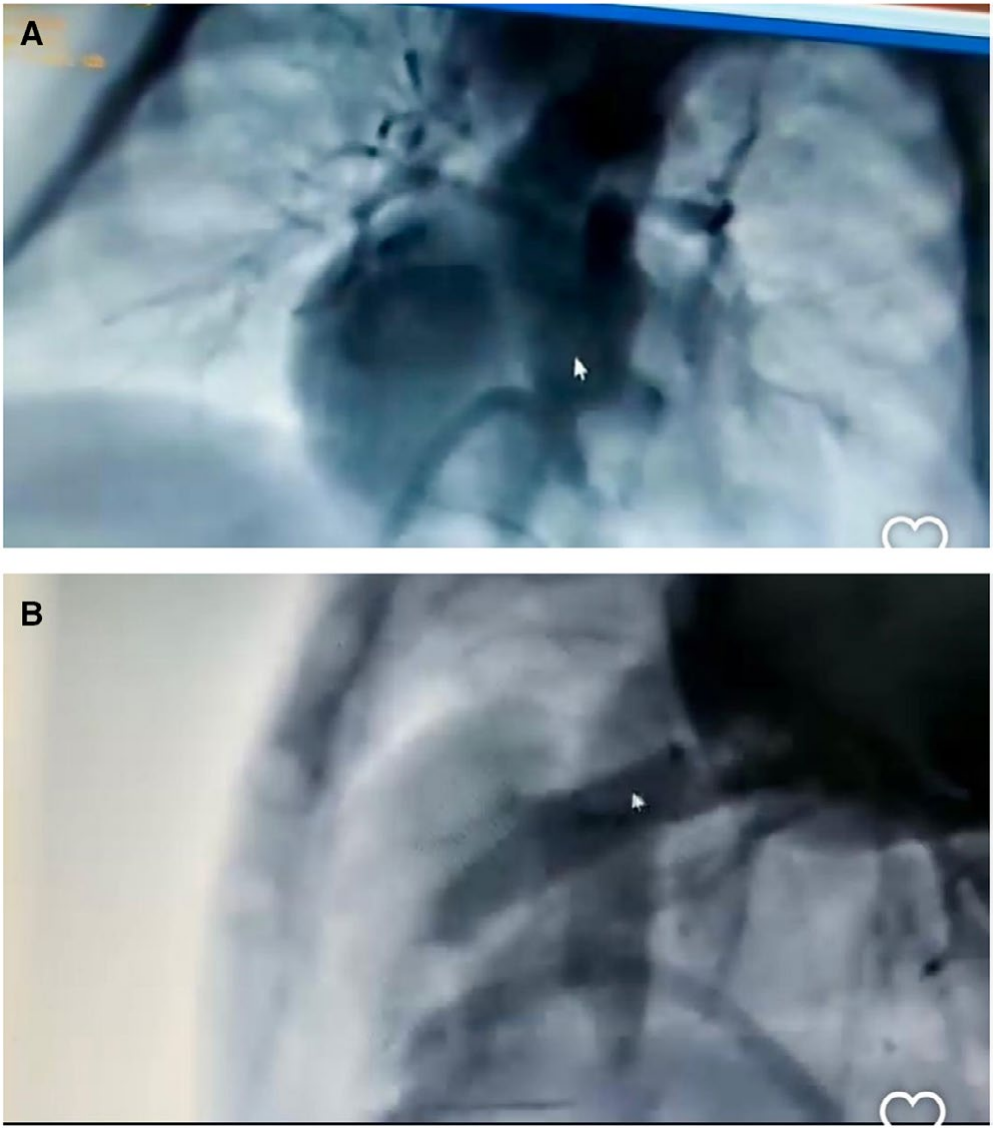
(A and B) Injection after stent opening, when RVOT stenosis has resolved and the peripheral blood supply has increased.

**FIGURE 3:**
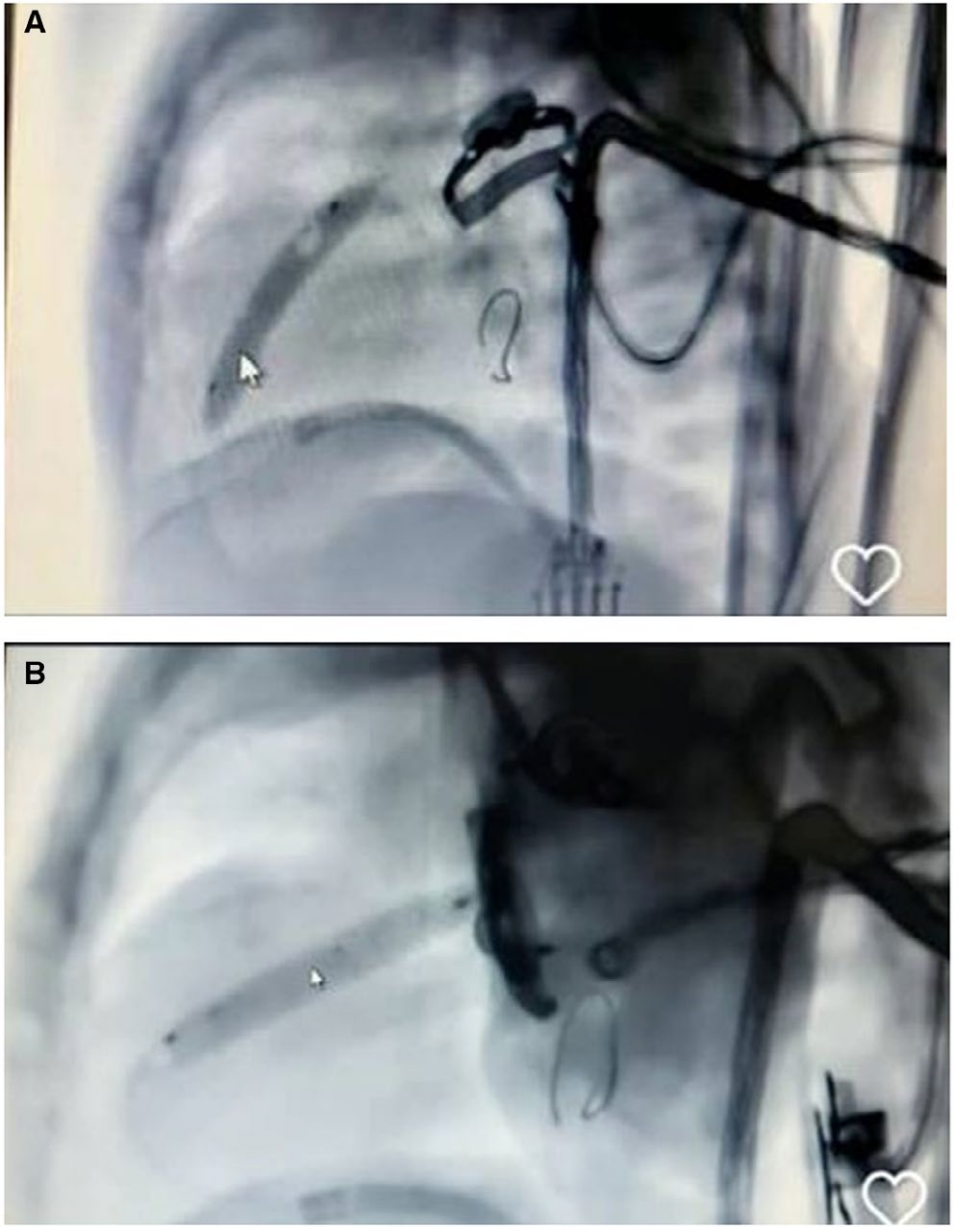
(A and B) Stenting steps.

Given persistent desaturation (SpO_2_ as low as 50%) and one CPR event, catheter‐based evaluation was pursued. On Day 4, after being established via femoral venous access, angiography confirmed severe RVOTO with tortuous anatomy and under‐perfused distal pulmonary arteries (Figure 1). A 4.5 mm Xience drug‐eluting coronary stent was positioned across the RVOT over a 0.014‐in. Grand Slam wire. Post‐deployment angiography demonstrated improved pulmonary perfusion and maintained SpO_2_ levels above 80% (Figures 2 and 3).

## 6 Post‐Procedure Management

Discontinued PGE_1_; started heparin infusion (5 U/kg/h).

Aspirin (7 mg/kg/day) and clopidogrel were initiated.

Weaned milrinone, dopamine, and propranolol over 2–3 days.

Blood cultures were negative (Table 1).

**TABLE 1:** Data obtained from the patient's laboratory examinations.

Index	Value at 2025/01/19	Value at 2025/03/10
White blood cell count	14.1	14.5
Hemoglobin	16 mg/dL	11 mg/dL
Platelet count	219,000	727,000
Na	140	136
K	5.1	4.6
Ca	4.1	4.4
Urea	28	17
Creatinine	1	0.5
	**Value at 2025/01/19**	**Value at 2025/01/24**
Blood cultures	No growth	No growth

## 7 Follow‐Up and Outcomes

The neonate was weaned from mechanical ventilation on Day 8 and transitioned to NIPPV. She developed bronchopulmonary dysplasia, managed with corticosteroids, and was successfully weaned to spontaneous breathing over the following month. On Day 51, she was discharged on aspirin and clopidogrel with SpO_2_ 82%–85%, stable, and scheduled for follow‐up and definitive surgery.

**FIGURE 4:**
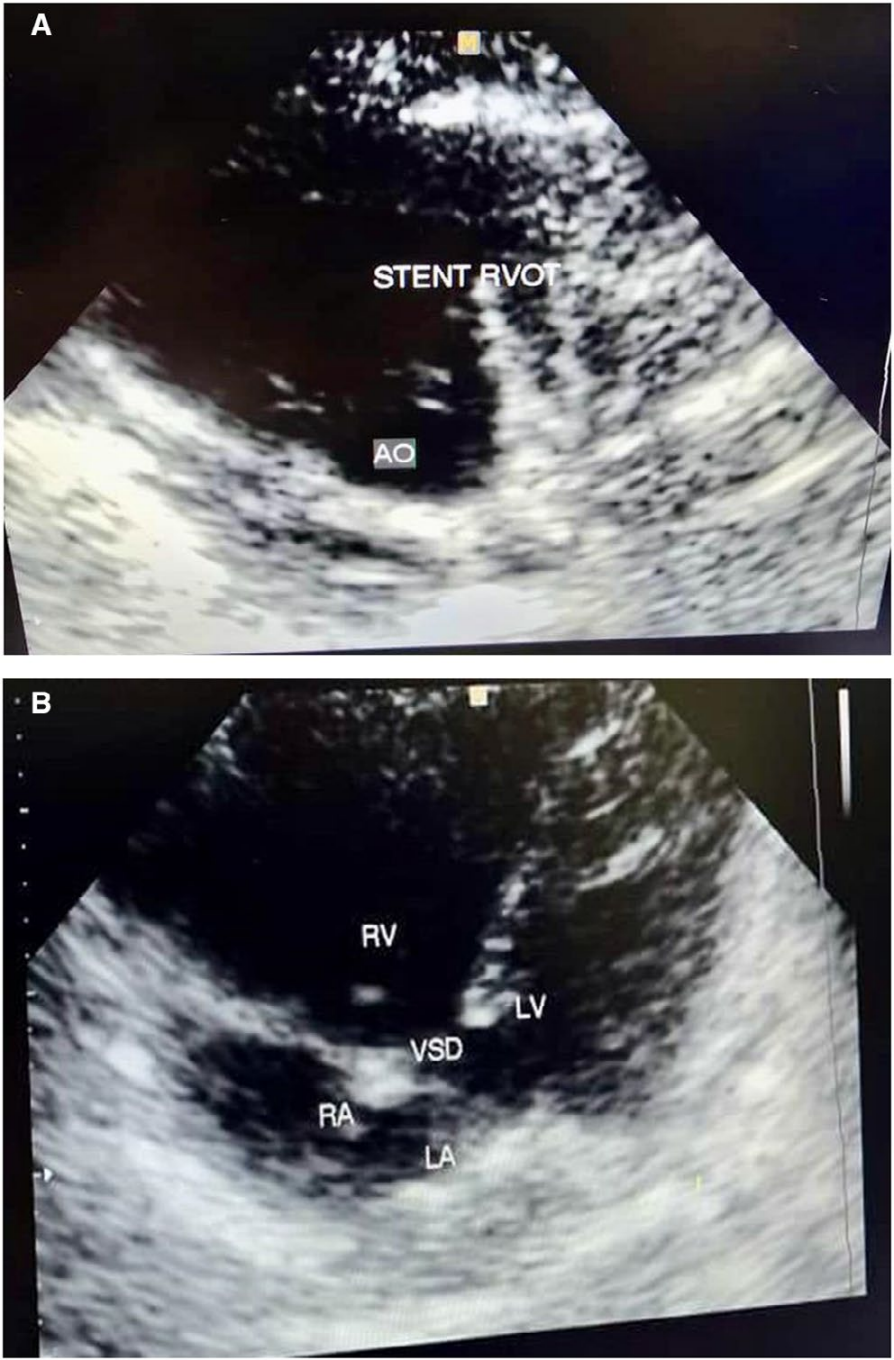
(A and B) Echocardiography after stent placement surgery.

Post‐stenting echocardiography showed improved antegrade flow across the RVOT, with increased perfusion and interval growth of the RPA and LPA to ~2.5–3.0 mm over the following weeks (Figure 4).

Given a history of two prior neonatal TOF‐associated deaths, genetic evaluation was recommended; the family declined testing.

**FIGURE 5:**
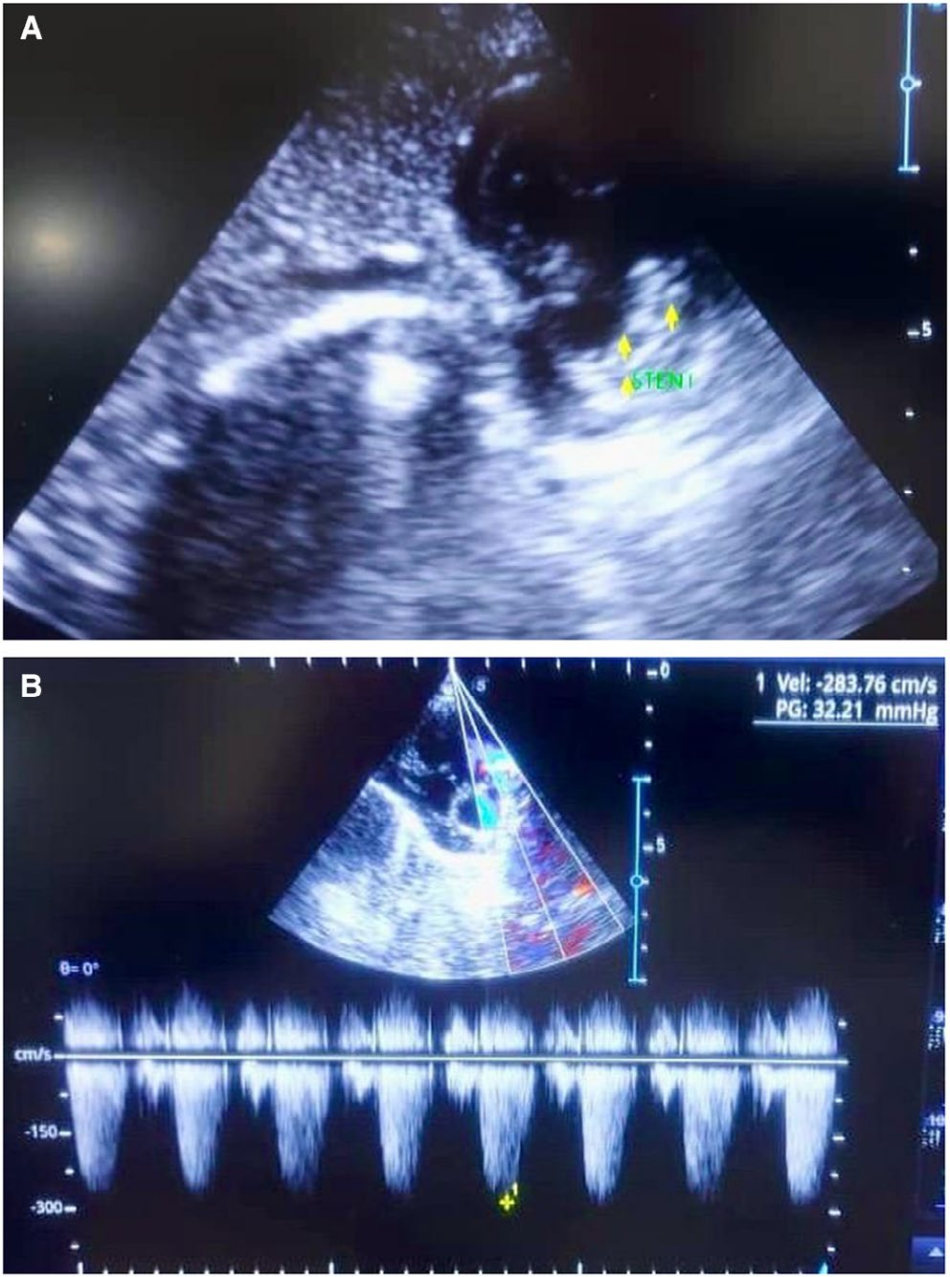
(A and B) Echocardiography 6 months after stent placement surgery.

After 6 months, the patient is gaining weight appropriately. An echocardiography was performed, and the RVOT stent is open; heart function is normal. The SpO_2_ is 80% (Figure 5).

## 8 Discussion

Preterm infants with TOF and critical RVOTO pose high perioperative risks. In such patients, RVOT stenting offers a minimally invasive bridge to defer definitive repair. In our case, these complexities were further compounded by a positive family history of TOF‐related mortality and a hypoplastic pulmonary artery anatomy [8].

Initial echocardiographic assessment within hours after birth revealed critical valvular and subvalvular pulmonary stenosis, near absence of antegrade pulmonary flow, severe bilateral branch pulmonary artery hypoplasia (1.5–2.0 mm), and severe tricuspid regurgitation. These findings indicated a functionally near‐atretic RVOT with tortuous morphology. Under such circumstances, the timely establishment of reliable pulmonary blood flow (PBF) is critical.

In this case, RVOT stenting with a coronary drug‐eluting stent (DES) provided immediate hemodynamic improvement, evidenced by increased oxygen saturation and echocardiographic confirmation of restored antegrade flow through the RVOT. Follow‐up imaging over the subsequent weeks demonstrated interval growth of the RPA and LPA, rising from approximately 1.5–2.0 to 2.5–3.0 mm, suggesting that stent patency contributed to improved pulmonary artery development [9].

These findings are consistent with prior studies reporting enhanced pulmonary arterial growth following RVOT stenting. Li et al. [10] demonstrated that RVOT stenting results in significantly greater and more symmetrical growth of both RPA and LPA compared to the modified Blalock–Taussig shunt (mBTS).

Our case confirms that drug‐eluting stents, by minimizing tissue reactivity and maintaining RVOT patency, offer reliable pulmonary blood flow support and facilitate more balanced pulmonary artery growth. Compared to surgical shunts, RVOT stenting may thus represent a superior palliative option in selected high‐risk neonates when early complete repair is not feasible [7].

Although some studies have highlighted complications such as stent migration, re‐intervention, or arrhythmias, the overall short‐term mortality remains low, and the procedure is generally safe and well‐tolerated [7, 11, 12, 13]. The post‐procedural outcome showed remarkable clinical improvement, with oxygen saturation rising above 80% and no procedural complications.

Advantages include rapid improvement in oxygenation and stabilization of pulmonary hemodynamics, facilitating growth before surgery. Most of the articles involved term or older infants with RVOT‐specific stents. The use of coronary stents in a premature infant on the fourth day after birth is a rare case report.

The strong family history raises suspicion of genetic syndromes, notably the 22q11.2 deletion (DiGeorge syndrome), despite normal calcium levels and the absence of dysmorphic features. Genetic evaluation should be pursued as part of long‐term management, although the family initially declined testing [14].

## 9 Limitations

Single case, limiting generalizability.

Short‐term follow‐up.

No genetic evaluation performed.

## 10 Conclusion

RVOT stenting using a drug‐eluting coronary stent appears to be a viable palliative option for preterm neonates with complex TOF and critical RVOTO. This approach provides life‐saving pulmonary perfusion, stabilizes the patient, and enables elective, definitive repair.

## Author Contributions

**Reza Derakhshan:** conceptualization, investigation, methodology, project administration, supervision, validation, visualization. **Mahsa Shadman:** data curation, formal analysis, investigation, methodology, resources, writing – original draft, writing – review and editing. **Najmeh Soltani Nejad:** conceptualization, data curation, investigation, methodology, project administration, resources, supervision, validation, visualization, writing – review and editing.

## Funding

The authors have nothing to report.

## Ethics Statement

The study was approved by the Ethics Committee of Kerman University of Medical Sciences (Ethics Code: IR.KMU.AH.REC.1404.160).

## Consent

Written informed consent was obtained from the patient's legal guardians for publication of this case report and any accompanying images and clinical information.

## Conflicts of Interest

The authors declare no conflicts of interest.

## Data Availability

All data generated or analyzed during this study is included in this published article.
